# Atractylenolide III Improves Mitochondrial Function and Protects Against Ulcerative Colitis by Activating AMPK/SIRT1/PGC-1*α*

**DOI:** 10.1155/2022/9129984

**Published:** 2022-04-08

**Authors:** Jieru Han, Wenhao Li, Guangyu Shi, Yunlei Huang, Xutao Sun, Na Sun, Deyou Jiang

**Affiliations:** ^1^Department of Synopsis of the Golden Chamber, School of Basic Medical Sciences, Heilongjiang University of Chinese Medicine, Harbin, Heilongjiang 150040, China; ^2^Department of Ultrasound Medicine, First Affiliated Hospital, Heilongjiang University of Chinese Medicine, Harbin, Heilongjiang 150040, China; ^3^Foundamental Training Base, Air Force Aviation University, Changchun, Jilin 130021, China; ^4^Department of Typhoid, School of Basic Medical Sciences, Heilongjiang University of Chinese Medicine, Harbin, Heilongjiang 150040, China; ^5^School of Basic Medical Sciences, Heilongjiang University of Chinese Medicine, Harbin, Heilongjiang 150040, China

## Abstract

Ulcerative colitis (UC) is a complex inflammatory bowel disease (IBD) associated with mitochondrial function. Atractylenolide III (AT III) is a natural product with anti-inflammatory effects. The aim of this work is to investigate the protective effect of AT III on UC and its underlying mechanisms. Herein, dextran sulfate sodium- (DSS-) induced mice and lipopolysaccharide- (LPS-) stimulated intestinal epithelial cells (IEC-6) were employed to mimic UC pathologies *in vivo* and *in vitro*. The results showed that in DSS-induced mice, AT III significantly reversed the body weight loss, colon length reduction, disease activity index (DAI) increase, and histological damage. The production of proinflammatory factors and reduction of antioxidants in colitis were suppressed by AT III. In addition, we demonstrated that AT III attenuated the intestinal epithelial barrier destruction and mitochondrial dysfunction induced by DSS, which was similar to the *in vitro* results in LPS-treated IEC-6 cells. The protein levels of p-AMPK, SIRT1, and PGC-1*α* along with acetylated PGC-1*α* were also upregulated by AT III *in vivo* and *in vitro*. In conclusion, these findings support that AT III may protect against mitochondrial dysfunction by the activation of the AMPK/SIRT1/PGC-1*α* signaling pathway during UC development.

## 1. Introduction

Ulcerative colitis (UC) is a chronic relapsing inflammatory bowel disease (IBD) caused by multiple factors, such as environment, gene, and immunoregulator [1, 2]. It is characterized that epithelial damage, neutrophil infiltration, microbial translocation, and inflammatory condition are important features of UC [3]. However, the pathogenesis of UC still needs to be further explored.

It is demonstrated that mitochondrial changes are critical hubs of cellular physiology during UC development [4]. For example, mitochondrial ultrastructural disruption and replication reduction are revealed in the lesions of UC [5, 6]. Mitochondrial electron transport chain complex activities are also altered in UC patients or mouse model [7, 8]. In addition, accumulating evidence suggests that epithelial barrier dysfunction might be attributed to mitochondrial abnormalities in colitis [9]. Ho et al. demonstrate that loss of MDR1 results in mitochondrial impairment along with increased mROS production driving epithelial barrier dysfunction in colitis [10]. It is well known that proliferator-activated receptor-*γ* coactivator 1-*α* (PGC-1*α*) is a key regulator of mitochondrial biogenesis and function [11]. Decreased PGC-1*α* in the intestinal epithelium of UC caused significant mitochondrial impairment, epithelial barrier damage, and inflammation [12]. Thus, it is necessary to illustrate the role of mitochondrial function in colitis development.

Atractylenolide III (AT III) is one of the main bioactive compounds from the root extracts of *Atractylodes macrocephala* Koidz, which has anti-inflammatory, neuroprotective, and gastroprotective properties [13–15]. Several studies show that AT III suppresses the production of TNF-*α*, iNOS, and IL-6 [16, 17]. Song et al. demonstrate that AT III maintains energy metabolism of skeletal muscle cells to protect against obesity and T2DM [18]. In addition, AT III is also reported to inhibit microglial mitochondrial fission in ischemic injury [19]. However, whether AT III protects against UC progression is unclear.

Dextran sodium sulfate- (DSS-) induced mouse is considered an ideal model due to its similarity to human UC in terms of physiology, anatomy, and immune system [20]. Lipopolysaccharide (LPS) is a known inducer of intestinal epithelial barrier dysfunction [21, 22]. Therefore, this present study was designed to explore the potential effects of AT III on experimental colitis and underlying mechanisms using DSS-induced mice *in vivo* and LPS-stimulated IEC-6 cells *in vitro*.

## 2. Materials and Methods

### 2.1. Animals

All animals were cared in compliance with the Guide for the Care and Use of Laboratory Animals. Ethical approval was obtained from the Animal Experimental Ethics Committee of the First Affiliated Hospital, Heilongjiang University of Chinese Medicine.

Eight-week-old C57BL/6J male mice (20-23 g) were purchased from Beijing HFK Bioscience Co., Ltd. (Beijing, China) and maintained in standard laboratory conditions with a 12 h light/dark cycle at 22 ± 1°C with 45-55% humidity. All mice had free access to obtain standard murine chow diet and sterile water. At the initiation of experiments, five mice in each cage were housed and adaptively fed for a week.

### 2.2. Induction of the UC Animal Model

Mice were randomly separated into five groups: control group, DSS group, DSS+SASP group, DSS+AT III (L) group, and DSS+AT III (H) group. In brief, mice received 3% DSS (160110, MP Biochemicals, Santa Ana, CA, USA) in drinking water for 7 consecutive days to establish UC models. For drug administration, DSS mice were injected with 5 mg/kg or 10 mg/kg AT III (A2987, Sigma-Aldrich, St. Louis, MO, USA) through the tail vein once a day for 7 days. Furthermore, mice in the DSS+SASP group were administrated with 200 mg/kg/day sulfasalazine (SASP; S129986, Aladdin, Shanghai, China) orally for 7 consecutive days. SASP was used as a positive control drug. Control mice received the same amount of water without DSS.

During the experiments, the bedding materials of all cages were changed simultaneously to minimize the effect of environmental factors. All animals were weighed and monitored for health signs daily. Also, stool consistency and rectal bleeding were recorded to evaluate the disease activity index (DAI) scores as described previously [23]. Finally, mice (*n* = 12 per group) were sacrificed by using 150 mg/kg pentobarbital sodium intraperitoneally, and colons were harvested for further investigations.

### 2.3. Hematoxylin-Eosin (H&E) Staining

Colons from mice were fixed in 4% paraformaldehyde, embedded in paraffin, and then cut into 5 *μ*m thick sections. The sections were stained with hematoxylin (H8070, Solarbio, Beijing, China) for 5 min and eosin (A600190, Sangon, Shanghai, China) for 3 min. Images were observed under a microscope (BX53, Olympus, Tokyo, Japan) at 100x magnification. The histological scores were assessed blindly to evaluate the level of colitis as described by Banerjee et al. [24].

### 2.4. Immunohistochemistry

An immunohistochemistry assay was carried out with paraffin sections as mentioned above. The deparaffinized sections were rehydrated in ethanol and heated in citrate buffer for 10 min for antigen retrieval. After blocking in goat serum (SL038, Solarbio) for 15 min at room temperature, the sections were probed with the primary antibody against myeloperoxidase (MPO; 1 : 50, A1374, ABclonal, Wuhan, China) at 4°C overnight, followed by the secondary antibody (1 : 500, #31460, Thermo Fisher, Pittsburgh, PA, USA) incubation for 60 min at 37°C. Slices were cultured in DAB reagent and counterstained with hematoxylin. Finally, tissue sections were viewed on a microscope at 400x magnification.

### 2.5. Determination of MDA and GSH Content and SOD Activity

Proteins from homogenized colonic tissues were quantified using a BCA protein assay kit (P0009, Beyotime, Shanghai, China) and prepared to determine the concentration of malondialdehyde (MDA; A003-1, Nanjing Jiancheng Bioengineering Institute, Nanjing, China) and glutathione (GSH; A006-2, Nanjing Jiancheng Bioengineering Institute), as well as the activity of superoxide dismutase (SOD; A001-1, Nanjing Jiancheng Bioengineering Institute).

### 2.6. Cell Culture and Treatment

Rat intestinal epithelial cell line IEC-6 cells (Zhong Qiao Xin Zhou Biotechnology Co., Ltd., Shanghai, China) were cultured in Dulbecco's modified Eagle medium (DMEM; SH30027, HyClone, Logan, UT, USA) containing 10 *μ*g/ml insulin and 10% fetal bovine serum (FBS; 04-011-1A, BI, Kibbutz Beit-Haemek, Israel) in an incubator with 5% CO_2_ at 37°C. Cells were treated with 50 *μ*g/ml LPS (L2630, Sigma-Aldrich) for 12 h to mimic the intestinal epithelial cell damage in UC. In addition, IEC-6 cells were treated with AT III (80 *μ*M) alone for 24 h or AT III (40 or 80 *μ*M) for 12 h prior to LPS treatment.

### 2.7. Intestinal Permeability Assay

For the *in vitro* permeability assay, cells were seeded in the upper chamber at the density of 5 × 10^5^ per well. After treatment, IEC-6 cell monolayers were cultured in 1 mg/ml FITC-dextran 20 (FD20, TdB Labs, Uppsala, Sweden). The solution (100 *μ*l) was collected from the basal chamber at 60, 120, 180, or 240 min, respectively, and the fluorescent intensity was measured.

### 2.8. JC-1 Assay

The mitochondrial membrane potential (MMP) of IEC-6 cells was determined using the JC-1 (5,5′,6,6′-Tetrachloro-1,1′,3,3′-tetraethylbenzimidazolylcarbocyanine iodide) assay. In brief, IEC-6 cells were incubated with 0.5 ml JC-1 staining working solution (C2006, Beyotime) at 37°C for 20 min. After washing in JC-1 buffer (1×), cells were collected to measure MMP using a flow cytometer (NoyoCyte, Aceabio, San Diego, CA, USA).

### 2.9. Determination of Complex I and Complex IV Activity

The colon tissues or IEC-6 cells were used to assess the activities of complex I and complex IV. Protein samples were extracted for quantification and then prepared to evaluate the activity of mitochondrial electron transport enzymes using the complex I activity assay kit (BC0510, Solarbio) and complex IV activity assay kit (BC0940, Solarbio).

### 2.10. Immunofluorescence

For the immunofluorescence assay, tissue sections were prepared as mentioned above, and cell slides were blocked in goat serum. Subsequently, colon sections or cell slides were incubated with primary antibodies against occludin (1 : 100; A2601, ABclonal), ZO-1 (1 : 100; AF5145, Affinity, Changzhou, China), or Tom20 (1 : 50; A18047, ABclonal) at 4°C overnight. Then, an FITC-labeled goat anti-rabbit IgG antibody (1 : 200; A0562, Beyotime) or Cy3-labeled goat anti-rabbit IgG antibody (1 : 200; A0516, Beyotime) was used to label tissue sections or cell slides at room temperature. After staining with DAPI (D106471, Aladdin), tissue sections or cell slides were observed using the microscope.

### 2.11. Quantitative Real-Time PCR

Total RNAs in cells or colonic tissues were isolated using the RNA simple total RNA kit (RP1201, BioTeke, Beijing, China) and quantified with Nano 2000 (Thermo Fisher). Then, the Super M-MLV Reverse Transcriptase (2641A, Takara, Beijing, China) was prepared to transcribe RNAs reversely into cDNAs. The quantitative real-time PCR (qPCR) analysis was carried out on an Exicycler™ 96 instrument (BIONEER, Daejeon, Korea) using the SYBR Green reagent (EP1602, BioTeke). Relative expression of mRNA was calculated using the 2^−ΔΔCT^ method and measured by the ratio of mRNA to *β*-actin. Primer sequences are listed in Table 1.

### 2.12. mtDNA quantification

Mitochondrial DNA (mtDNA) from colon tissues or IEC-6 cells was extracted with a mitochondrial DNA isolation kit (K280-50, BioVision, Milpitas, CA, USA). The specific primers are listed in Table 1. Relative quantification of mtDNA was measured by the ratio of mtDNA to *β*-globin using qPCR analysis.

### 2.13. Western Blot

Whole cell lysates were extracted using the RIPA lysate (P0013B, Beyotime) and quantified with a BCA protein assay kit. Equal amounts of protein samples were separated by SDS-PAGE and transferred onto the PVDF membrane. After blocking in 5% BSA, membranes were incubated with primary antibodies overnight at 4°C. Subsequently, immunoblots were incubated with secondary antibodies for 40 min at 37°C and visualized using Western ECL Substrate (E003, 7 Sea Biotech, Shanghai, China).

All antibodies were as follows: Occludin (1 : 1000; A2601, ABclonal), ZO-1 (1 : 1000; A0659, ABclonal), PGC-1*α* (1 : 1000; A17089, ABclonal), NRF-1 (1 : 1000; A5547, ABclonal), NRF-2 (1 : 1000; A0674, ABclonal), Tfam (1 : 1000; A13552, ABclonal), AMPK (1 : 1000; AF6423, Affinity), p-AMPK (1 : 1000; AF3423, Affinity), SIRT1 (1 : 1000; A11267, ABclonal), *β*-actin (1 : 2000; 60008-1-Ig, Proteintech, Wuhan, China), goat anti-mouse IgG antibody (1 : 10000; SA00001-1, Proteintech), and goat anti-rabbit IgG antibody (1 : 10000; SA00001-2, Proteintech).

### 2.14. Immunoprecipitation Assay

The extracted proteins were incubated with 1 *μ*g PGC-1*α* antibody (sc-518025, Santa Cruz, USA) overnight at 4°C and then incubated with Protein A Agarose beads at 4°C for 2 h. After that, the immunoprecipitates were collected and subjected to SDS-PAGE for further Western blot analysis with specific antibodies: acetyl lysine antibody (1 : 1000; DF7729, Affinity) and PGC-1*α* antibody (1 : 1000; A17089, ABclonal).

### 2.15. Statistical Analysis

Data were expressed as mean ± standard deviation (SD) and analyzed by using GraphPad Prism 8.0. One-way or two-way repeated analysis of variance (ANOVA) followed by Bonferroni's multiple comparison test was used to assess the statistical significance among multiple groups. The difference among the groups for the histological score was determined using the Kruskal-Wallis test following Dunn's multiple comparison test. *p* < 0.05 was considered statistically significant.

## 3. Results

### 3.1. AT III Protects against DSS-Induced UC in Mice

**Figure 1:**
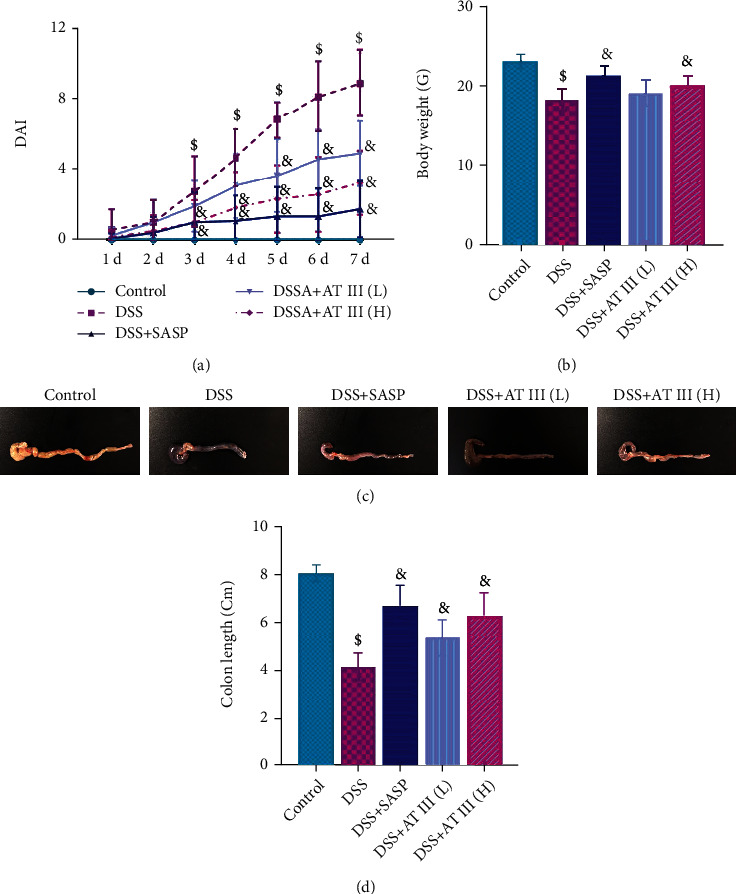
AT III protects against the symptoms of DSS-induced UC. (a) DAI scores. (b) Body weight. (c, d) Colon length. Scale bar: 1 cm. ^$^*p* < 0.05, compared to control; ^&^*p* < 0.05, compared to DSS.

DSS-induced mice were established to explore the effect of AT III on UC. As shown in Figure 1(a), mice challenged with DSS exhibited significant increase in DAI scores from the 3^rd^ day, which was recovered by AT III or SASP treatment. Administration of AT III or SASP also reversed the reduction of body weight and colon length caused by DSS (Figures 1(b)–1(d)). The mitigated rectal bleeding was observed in UC mice treated with AT III or SASP from the macroscopical images of colons (Figure 1(c)). These results indicate the beneficial effect of AT III on UC progression.

### 3.2. AT III Ameliorates DSS-Induced Inflammation and Oxidative Stress

**Figure 2:**
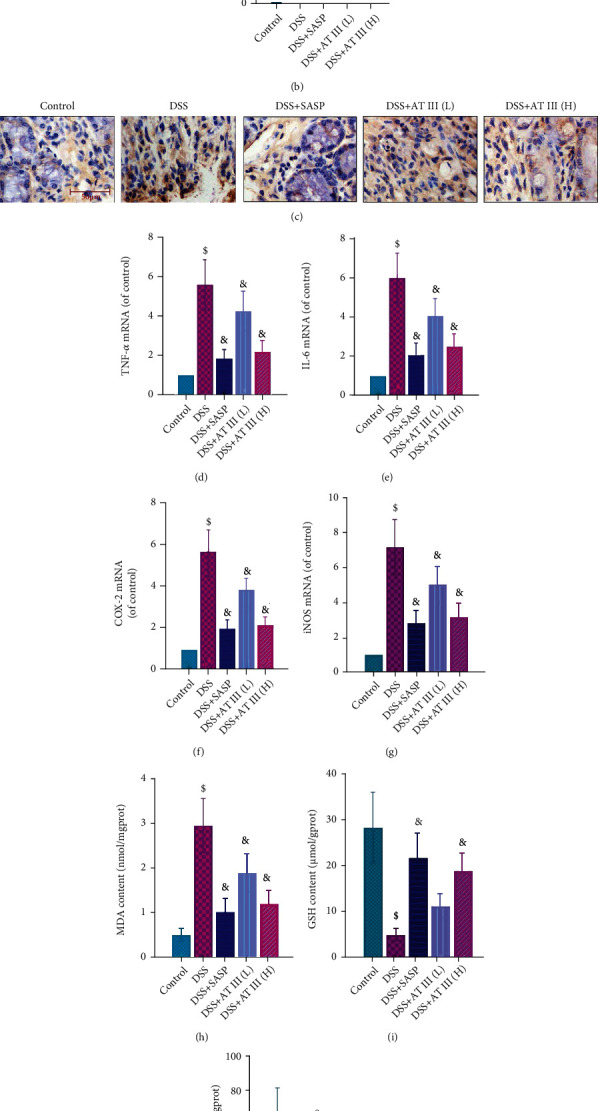
AT III ameliorates DSS-induced inflammation and oxidative stress. (a, b) H&E staining of colon tissues and quantification of histological scores. Scale bar: 200 *μ*m. (c) Immunohistochemical detection of MPO in colons. Scale bar: 50 *μ*m. (d–g) qPCR results of TNF-*α*, IL-6, COX-2, and iNOS mRNA in colons. (h–j) ELISA measurements of MDA and GSH content, as well as SOD activity. ^$^*p* < 0.05, compared to control; ^&^*p* < 0.05, compared to DSS.

Inflammation and oxidative stress are major pathological changes in UC. Histological results demonstrated that AT III or SASP treatment significantly alleviated the crypt damage, inflammatory cell infiltration, and goblet cell loss in colons of DSS-treated mice (Figures 2(a) and 2(b)). The MPO levels in colon tissues of UC mice were also reduced by AT III or SASP, which are shown in Figure 2(c). In addition, we noticed that the AT III or SASP treatment significantly inhibited the upregulation of proinflammatory factors in colons of UC mice, including TNF-*α*, IL-6, COX-2, and iNOS (Figures 2(d)–2(g)). The increase in MDA levels, reduction of GSH concentration, and inactivation of SOD activity in colons of DSS-treated mice were also significantly reversed by AT III or SASP (Figures 2(h)–2(j)). Thus, the data suggest that AT III might attenuate the inflammation and oxidative stress in UC development.

### 3.3. AT III Inhibits DSS-Induced Intestinal Barrier Impairment

**Figure 3:**
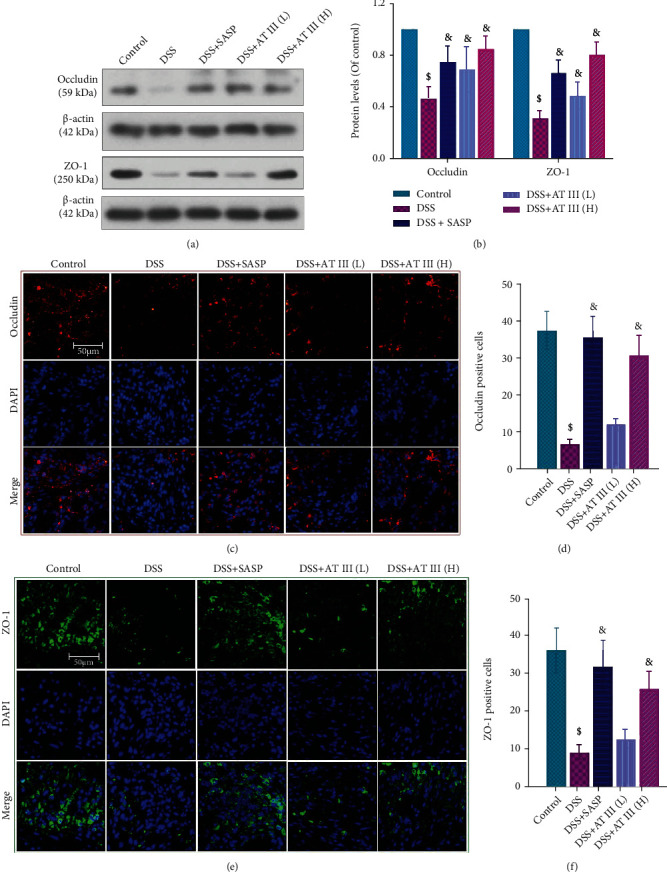
AT III inhibits DSS-induced intestinal barrier impairment. (a, b) Western blots for occludin and ZO-1 proteins and quantification results. (c, d) Representative immunofluorescent images for occludin and quantification results. (e, f) Representative immunofluorescent images for ZO-1 and quantification results. Scale bar: 50 *μ*m.

Considering that epithelial barrier damage is an essential event during UC development, we focused on investigating the effects of AT III on the intestinal epithelium. Western blot results showed that the decreased protein levels of occludin and ZO-1 in colons of UC mice were reversed by AT III or SASP (Figures 3(a) and 3(b)). Similar changes for occludin and ZO-1 in colons were also demonstrated by the immunofluorescence staining assay (Figures 3(c)–3(f)). It indicates the protective effect of AT III on intestinal barrier destruction in UC.

### 3.4. AT III Attenuates DSS-Induced Mitochondrial Dysfunction via AMPK/SIRT1-Mediated Deacetylation of PGC-1*α*

**Figure 4:**
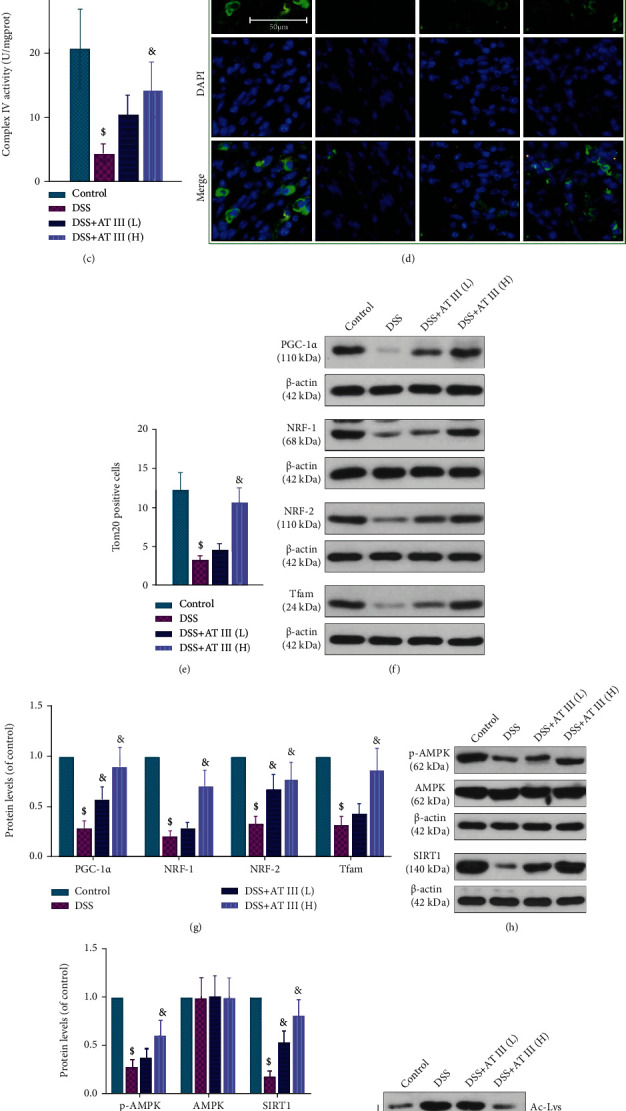
AT III attenuates DSS-induced mitochondrial dysfunction via AMPK/SIRT1-mediated deacetylation of PGC-1*α*. (a) The levels of mtDNA were determined using qPCR. (b, c) The activities of complex I and complex IV were measured using commercial kits. (d, e) Representative images of Tom20 expression using immunofluorescent staining and quantification results. Scale bar: 50 *μ*m. (f, g) The expression levels of PGC-1*α*, NRF-1, NRF-2, and Tfam were examined and quantified by Western blot. (h, i) The expression levels of p-AMPK, AMPK, and SIRT1 were measured and quantified by Western blot. (j, k) The acetylated levels of PGC-1*α* and quantification results were measured. ^$^*p* < 0.05, compared to control; ^&^*p* < 0.05, compared to DSS.

Mitochondrial dysfunction is a critical contributor to inflammation, oxidative stress, and barrier destruction. Thus, the effect of AT III on mitochondrial dysfunction in UC is focused in this study. The results showed that AT III treatment significantly upregulated the number of mtDNA copies and the activities of complex I and complex IV in colons of UC mice (Figures 4(a)–4(c)). The mitochondrial outer membrane protein Tom20 was also increased by AT III (Figures 4(d) and 4(e)). In addition, we observed that AT III significantly reversed the decreased expressions of mitochondrial-related proteins in colons of UC mice, including PGC-1*α*, NRF-1, NRF-2, and Tfam (Figures 4(f) and 4(g)).

The AMPK/SIRT1 signaling pathway is found to be the upstream of PGC-1*α*, and SIRT1 activates PGC-1*α* through NAD^+^-dependent deacetylation. However, whether the AMPK/SIRT1 signaling pathway mediates the effect of AT III on mitochondrial function is unclear. We demonstrated that the reduction in p-AMPK and SIRT1 protein levels in colons of DSS-treated mice was reversed by AT III (Figures 4(h) and 4(i)). In addition, AT III inhibited DSS-induced acetylation of PGC-1*α* in colons (Figures 4(j) and 4(k)). These results indicate that AT III attenuates mitochondrial dysfunction by activating AMPK/SIRT1-mediated deacetylation of PGC-1*α* to protect against UC.

### 3.5. AT III Abrogates LPS-Induced Intestinal Barrier Impairment in IEC-6 Cells

**Figure 5:**
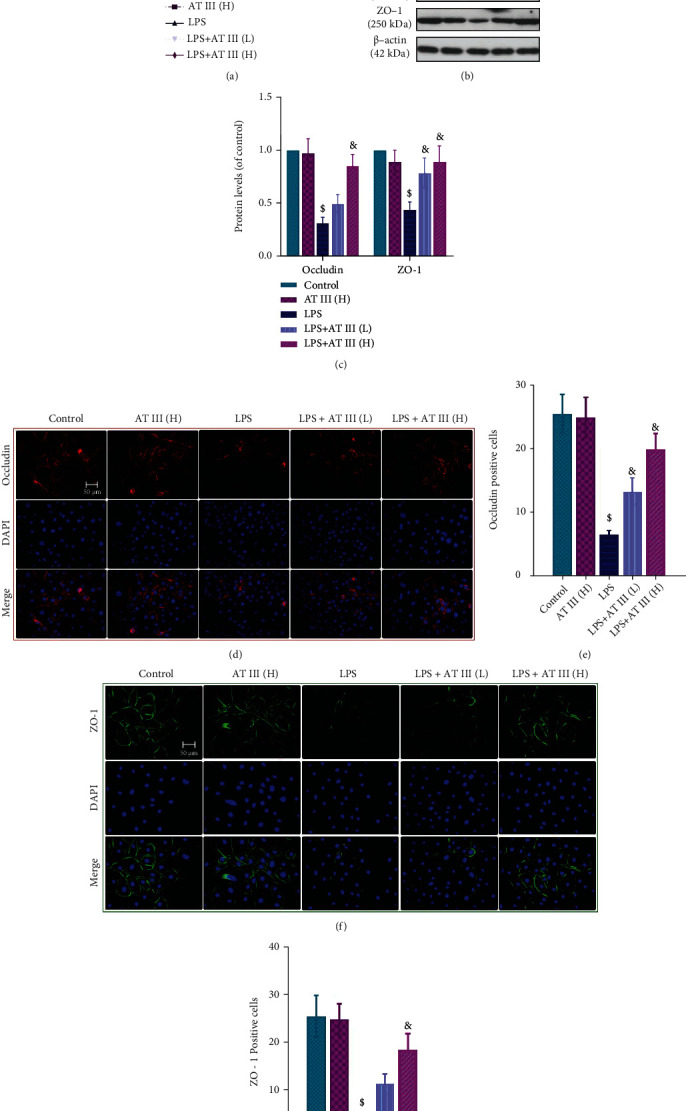
AT III abrogates LPS-induced epithelial barrier impairment in IEC-6 cells. (a) Paracellular permeability was measured using FITC-dextran. (b, c) Western blots for occludin and ZO-1 proteins and quantification results. (d, e) Representative images of occludin were determined and quantified by immunofluorescence. (f, g) Representative images of ZO-1 were measured and quantified by immunofluorescence. Scale bar: 50 *μ*m. ^$^*p* < 0.05, compared to control; ^&^*p* < 0.05, compared to LPS.

To further explore the effect of AT III on epithelial barrier function, LPS-treated IEC-6 cells were employed as an *in vitro* model. As shown in Figure 5(a), AT III effectively recovered the high cell permeability in LPS-stimulated IEC-6 cells. The levels of occludin and ZO-1 proteins in LPS-treated cells were increased by AT III treatment, as evidenced by both Western blot analysis (Figures 5(b) and 5(c)) and immunofluorescence staining results (Figures 5(d)–5(g)). These *in vitro* experiments confirm that AT III mitigates intestinal epithelial barrier damage induced by LPS.

### 3.6. AT III Attenuates LPS-Induced Mitochondrial Dysfunction in IEC-6 Cells via AMPK/SIRT1-Mediated Deacetylation of PGC-1*α*

**Figure 6:**
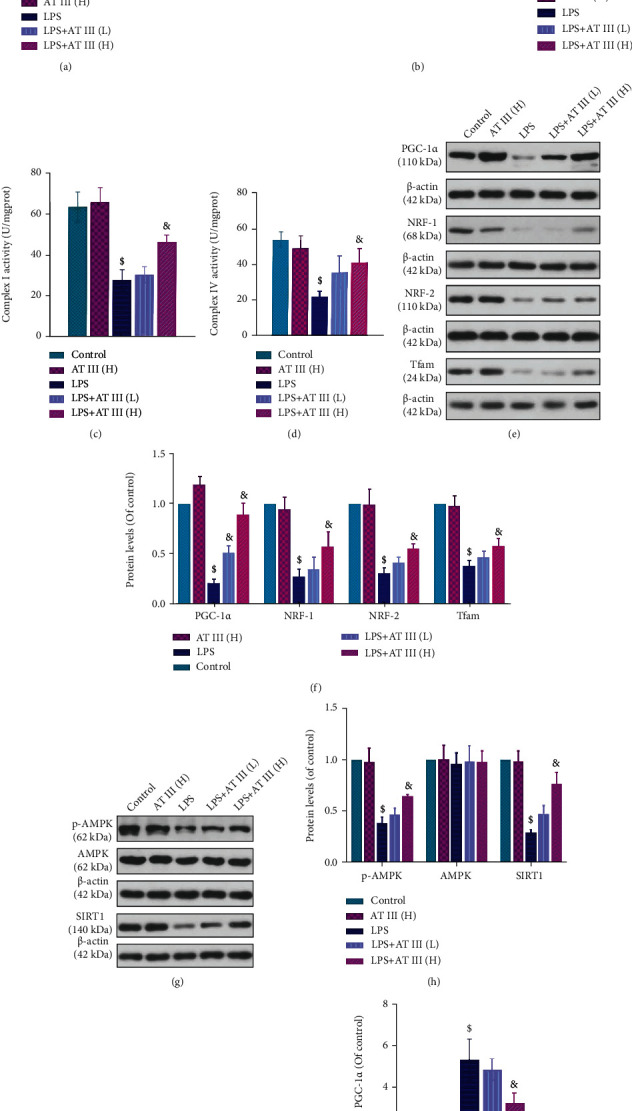
AT III attenuates LPS-induced mitochondrial dysfunction in IEC-6 cells via AMPK/SIRT1-mediated deacetylation of PGC-1*α*. (a) The levels of mtDNA were examined by qPCR. (b) The MMP changes were measured and quantified by the flow cytometry assay using JC-1 probes. (c, d) The complex I and complex IV activities were measured. (e, f) The protein expression levels of PGC-1*α*, NRF-1, NRF-2, and Tfam were detected and quantified by Western blot. (g, h) The protein expression levels of p-AMPK, AMPK, and SIRT1 were examined and quantified by Western blot. (i, j) The acetylated levels of PGC-1*α* and quantification results were determined. ^$^*p* < 0.05, compared to control; ^&^*p* < 0.05, compared to LPS.

**Figure 7:**
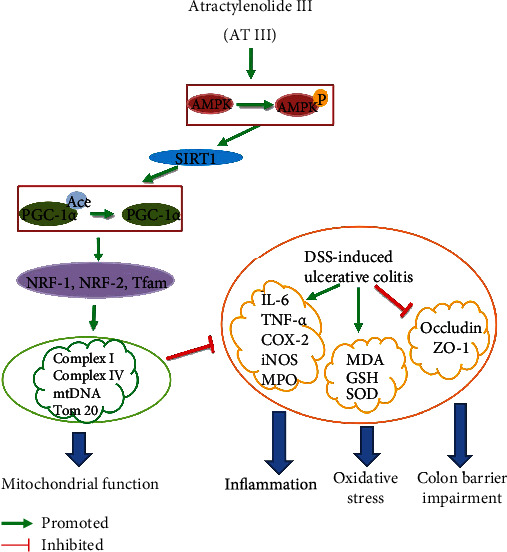
The mechanism of AT III in the amelioration of UC.

To assess the underlying mechanisms of AT III in the regulation of epithelial barrier function, mitochondrial function was further investigated *in vitro*. Similar to the alterations *in vivo*, decreased mtDNA copy number in LPS-treated cells was reversed by AT III (Figure 6(a)). The JC-1 staining assay showed that AT III reversed LPS-induced decrease in MMP in IEC-6 cells (Figure 6(b)). The reduction of electron transport chain complex activities and decreased expression of mitochondrial proteins in LPS-treated IEC-6 cells were also upregulated by AT III (Figures 6(c)–6(f)). In addition, we suggested that AT III significantly activated p-AMPK and SIRT1 protein levels and deacetylated PGC-1*α* in LPS-treated cells (Figures 6(g)–6(j)). Thus, these data imply that AT III might activate AMPK/SIRT1-mediated deacetylation of PGC-1*α* to attenuate mitochondrial dysfunction of intestinal epithelial cells, which has been summarized in Figure 7.

## 4. Discussion

Although a number of therapeutics are available for the treatment of IBD in a clinical setting, the significant side effects are heavy burden on the quality of life. Thus, this study was aimed at exploring a potential drug for the treatment of UC. Our results showed that AT III mitigated colitis symptoms, inhibited inflammation and oxidative stress, and restored epithelial barrier destruction in colons of UC mice. Experiments *in vivo* and *in vitro* suggested that AT III protected against mitochondrial dysfunction of the intestinal epithelium through the activation of AMPK/SIRT1/PGC-1*α*.

Inflammatory responses and oxidative stress are known to be important features of UC pathogenesis [25, 26]. The proinflammatory cytokines (such as TNF-*α*, IL-1*β*, and IL-6) and proteins (such as iNOS and COX-2) are important mediators of the inflammatory process in UC [27, 28]. MPO production is a key biomarker of activated neutrophils in IBD and is associated with oxidative stress [29]. Several reports showed that AT III suppressed the expression levels of proinflammatory factors (TNF-*α*, IL-6, and IL-1*β*) and oxidative stress factors (SOD and MDA) *in vivo* and *in vitro* [19, 30]. Consistent with these findings, our results demonstrated that AT III suppressed the inflammation and oxidative stress in colons of UC, suggesting a protective effect of AT III on experimental colitis.

Impaired structural and functional integrity of the epithelial barrier is discovered to exacerbate the intestinal inflammatory response and is correlated with the expression of tight junction proteins (including ZO-1 and occludin) [31–33]. In DSS-induced colitis mice, the significant intestinal barrier disruption and mucosal hyperpermeability were observed [34, 35]. Bein et al. also reported that LPS was a commonly used stimulator to cause hyperpermeability and barrier destruction in intestinal inflammatory disorders [36]. In this study, we found that AT III increased low levels of tight junction proteins and reduced hyperpermeability *in vivo* and *in vitro*. Thus, it indicates that the protective effect of AT III in colitis might be attributed to maintaining the barrier function of the intestinal epithelium.

Mitochondria are intracellular double-membrane-bound organelles that play a key role in inflammatory diseases such as rheumatoid arthritis and UC [37, 38]. The maintenance of mitochondrial function might counteract the inflammation, oxidative stress, and epithelial barrier damage in colitis [39, 40]. The *in vitro* experiments showed that LPS might lead to the significant intestinal injury and mitochondrial dysfunction [22]. However, whether mitochondrial function is regulated by AT III in the protection of the intestinal epithelial barrier remains unclear. Our results suggested that AT III attenuated mitochondrial dysfunction in colons of DSS-induced mice, which was similar to the results in LPS-treated IEC-6 cells. This finding was consistent with the finding that AT III suppressed mitochondrial dysfunction in microglia reported by Zhou et al. [19]. Interestingly, Boyapati et al. revealed an increased level of mtDNA in the plasma of UC patients [41], which was contrary to our results *in vivo* and *in vitro*. One possible explanation might be that during the severe tissue or cell injury, massive amounts of mtDNAs were released into the circulating plasma and exacerbated the inflammatory diseases. Although AT III has been shown to attenuate epithelial barrier disruption through maintaining mitochondrial function, its potential pathway still requires more investigations.

PGC-1*α*, an important regulator of mitochondrial biogenesis and function, has been shown to interact with nuclear respiration factors (NRF-1 and NRF-2) to activate Tfam in mtDNA replication/transcription [42, 43]. In addition, PGC-1*α* might be activated by AMPK and SIRT1 and deacetylated by SIRT1 in a NAD^+^-dependent manner [44, 45]. In experimental colitis, the enhanced PGC-1*α* deacetylation was shown to repair damaged mitochondria and maintain intestinal barrier function [12]. Our *in vivo* and *in vitro* results suggested that AT III increased PGC-1*α* expression and promoted its deacetylation through the activation of AMPK/SIRT1, which was in accord with the reports in skeletal muscle cells by Song et al. [18]. Altogether, it indicates that AMPK/SIRT1/PGC-1*α* may be a potential pathway mediating the protective effect of AT III on mitochondrial dysfunction in the intestinal epithelium of experimental colitis.

To explore the action mechanism of drugs or natural products for UC prevention or treatment, many chemicals are widely accepted to induce UC models, such as DSS, 2,4,6-trinitrobenzenesulfonic acid (TNBS), and oxazolone (OXA) [46]. In particular, DSS is the most common chemical for UC induction due to its availability, practicality, and reproducibility, and it is most similar to human UC in terms of clinical, histological, and immunophysiological aspects [47]. Due to the complex etiologies of UC, one of the limitations of this study is that DSS-induced experimental colitis does not fully cover the pathology of human UC. More experimental models will be used in the future to investigate the effect of AT III in UC and its mechanisms.

In conclusion, this present work suggests that AT III protects against mitochondrial dysfunction and ameliorates colitis development by the activation of the AMPK/SIRT1/PGC-1*α* signaling pathway, which is summarized in Figure 7. It highlights an important role of AT III for the treatment of UC.

## Figures and Tables

**Table 1:** Primer sequences of targeted genes.

Name	Sequences	Product length (bp)
MUS TNF-*α* F	CAGGCGGTGCCTATGTCTCA	182
MUS TNF-*α* R	GCTCCTCCACTTGGTGGTTT	
MUS IL-6 F	ATGGCAATTCTGATTGTATG	212
MUS IL-6 R	GACTCTGGCTTTGTCTTTCT	
MUS COX-2 F	AAAACCTCGTCCAGATGCTA	100
MUS COX-2R	TTGAGGAGAACAGATGGGAT	
MUS iNOS F MUS iNOS R	TTGGAGCGAGTTGTGGATTG GTGAGGGCTTGGCTGAGTGA	125
MUS *β*-actin F	CTGTGCCCATCTACGAGGGCTAT	155
MUS *β*-actin R	TTTGATGTCACGCACGATTTCC	
MUS mtDNA F MUS mtDNA R	GCCCATGACCAACATAACTG CCTTGACGGCTATGTTGATG	81
MUS *β*-globin F	AGGCAGAGGCAGGCAGAT	105
MUS *β*-globin R	GGCGGGAGGTTTGAGACA	
RAT mtDNA F RAT mtDNA R	ACACCAAGGTTAATGTAGC TTGAATCCATCTAAGCATT	62
RAT *β*-globin F	CAGTACTTTAAGTTGGAAACG	81
RAT *β*-globin R	ATCAACATAATTGCAGAGC	

## Data Availability

The raw data supporting the conclusions of this article will be made available by the authors, without undue reservation, to any qualified researcher.
